# Plasmon Resonance of Silver Nanoparticles as a Method of Increasing Their Antibacterial Action

**DOI:** 10.3390/antibiotics7030080

**Published:** 2018-08-22

**Authors:** Alexander Yu. Vasil’kov, Ruslan I. Dovnar, Siarhei M. Smotryn, Nikolai N. Iaskevich, Alexander V. Naumkin

**Affiliations:** 1 Nesmeyanov Institute of Organoelement Compounds, Russian Academy of Sciences, 28 Vavilov st., Moscow 119991, Russia; alexandervasilkov@yandex.ru (A.Y.V.); naumkin@ineos.ac.ru (A.V.N.); 2 Grodno State Medical University, 80 Gorky St., Grodno 230009, Belarus; s.smotrin@mail.ru (S.M.S.); inngrno@mail.ru (N.N.I.)

**Keywords:** antibacterial effect, laser irradiation, metal-vapour method, silver nanoparticles, TEM, XPS, EXAFS

## Abstract

In this article, a series of silver-containing dressings are prepared by metal-vapor synthesis (MVS), and their antibacterial properties are investigated. The antibacterial activity of the dressings containing silver nanoparticles (AgNPs) against some Gram-positive, and Gram-negative microorganisms (*Staphylococcus aureus*, *Staphylococcus haemolyticus*, *Pseudomonas aeruginosa*, *Klebsiella pneumoniae*, *Escherichia coli*, *Moraxella* spp.) has been determined. Based on the plasmon resonance frequency of these nanoparticles, the frequency of laser irradiation of the dressing was chosen. The gauze bandage examined showed pronounced antibacterial properties, especially to *Staphylococcus aureus* strain. When 470 nm laser radiation, with a power of 5 mW, was applied for 5 min, 4 h after inoculating the Petri dish, and placing a bandage containing silver nanoparticles on it, the antibacterial effect of the latter significantly increased—both against Gram-positive and Gram-negative microorganisms. The structure and chemical composition of the silver-containing nanocomposite were studied by transmission electron microscopy (TEM), X-ray photoelectron spectroscopy (XPS) and extended X-ray absorption fine structure (EXAFS). The synthesized AgNPs demonstrate narrow and monomodal particle size distribution with an average size of 1.75 nm. Atoms of metal in Ag/bandage system are mainly in Ag^0^ state, and the oxidized atoms are in the form of Ag-Ag-O groups.

## 1. Introduction

The widespread use of antibiotics for the treatment and prevention of bacterial diseases leads to a significant increase in the antibacterial resistance of microorganisms through its acquisition either through exogenous resistance genes or chromosomal mutations [1]. This stimulates not only the search for new antibacterial drugs, but also possible alternatives to the latter [2,3]. The scientists are tasked to find substances that effectively act simultaneously on Gram-positive, Gram-negative microorganisms and fungi, which are independent of the antibiotic resistance of the microorganism.

Silver and its compounds have been used in medicine since ancient times. Mass application of silver preparations began in the seventies of the 19th century [4,5]. Since then, numerous confirmations of antiviral, antibacterial, and immunomodulating activity of silver preparations have been received [6]. With the invention of antibiotics, which have more pronounced antibacterial properties, interest in the therapeutic properties of silver dramatically decreased. However, widespread use of antibiotics revealed by the end of the 20th century a number of their shortcomings and silver preparations were again actively studied and used [7].

The mechanism of antimicrobial effect of silver was carefully studied previously. It has now been established that silver ions are selectively toxic with respect to prokaryotic microorganisms with a weak effect on eukaryotic cells [6], including comparatively minimal toxicity to mammalian cells [8]. This is due to the fact that the concentration of silver ions or silver nanoparticles necessary for the death of prokaryotic cells (bacteria) is much lower than the concentration that causes the death of eukaryotic cells, including cells of the human body [9].

It should be noted that new prospects for the use of silver in medicine are opened in connection with the development of nanotechnology, an interdisciplinary field of science that deals with the creation, production and application of structures, devices and systems ranging in size from 1 to 1000 nm, although in practice they use the sizes ranging from 1 to 100 nm more often [10]. It is proved that the metal nanoparticles have unique properties, often differing from that of the solid metal [10]. This is due to the fact that the surface/bulk energy ratio of nanoparticles is much larger than that of compact metal [11].

As applied to medicine, this means that the nature of the interaction of a nanoparticle with a bacterium or fungus is significantly different from the impact of a compact metal on them and probably enhances their bactericidal or fungicidal activity [10].

Localized surface plasmon resonance is an optical phenomenon that is generated when light interacts with conductive nanoparticles that are smaller than the incident wavelength [12]. From the point of view of antibacterial properties of (silver nanoparticles) AgNPs, it is interesting to study how these properties change when plasmon resonance effect occurs.

One of the ecological and effective methods for producing mono- and bi-metallic nanoparticles and materials based on them is the metal-vapor synthesis (MVS). The method was used for preparation of silver-containing composite materials for medical applications [13]. It is assumed that MVS will be effective for the modification of wound dressings prepared from natural or synthetic materials with AgNPs.

In this regard, the wide introduction of dressings containing AgNPs correctly combined with the plasmon resonance effect can play a significant role in improving treatment of purulent wounds in the era of increasing antibiotic resistance of microorganisms.

The aim of this research is to study the antibacterial effect of a new dressing material, based on gauze bandage containing AgNPs prepared by MVS, and changing this effect under the influence of laser radiation.

## 2. Results

The structure and chemical composition of the silver-containing nanocomposite were studied by transmission electron microscopy (TEM), X-ray photoelectron spectroscopy (XPS) and extended X-ray absorption fine structure (EXAFS).

**Figure 1:**
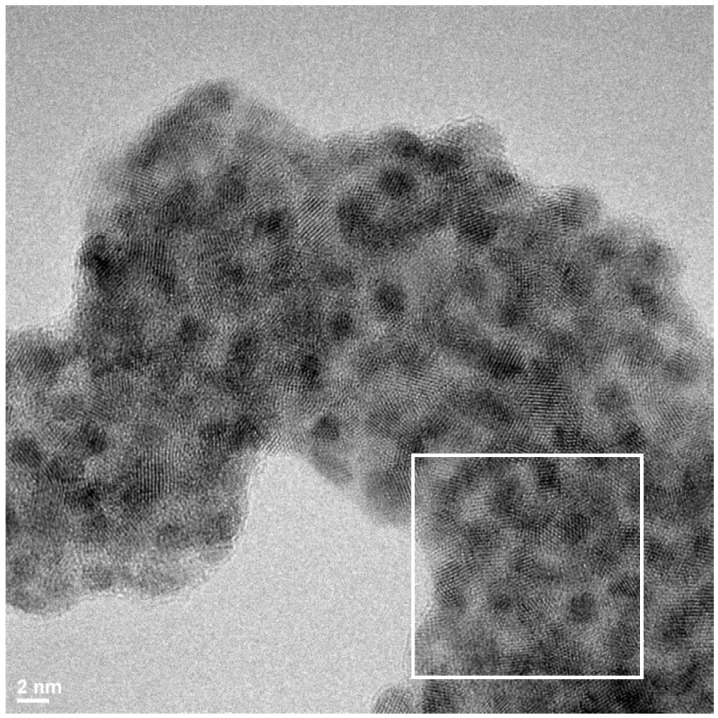
Transmission electron microscopy (TEM) micrograph of Ag-cotton system.

**Figure 2:**
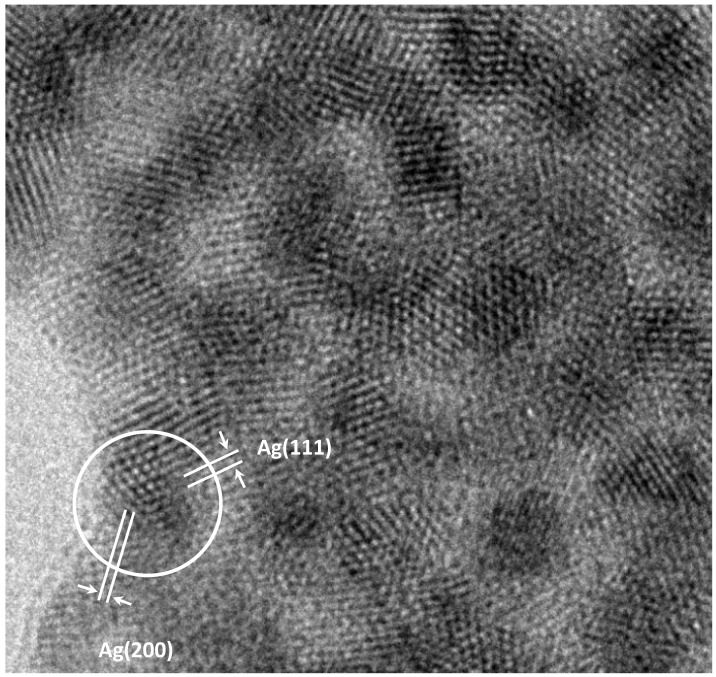
An enlarged image of the regions of ordered atoms on a surface, marked with a square in Figure 1.

The TEM micrograph of the cotton fibre is shown in Figure 1. The photograph shows amorphous extended structure with a diameter in the range of 16–25 nm, and dark crystalline nanostructures, the density of which is higher than that of cotton. The enlarged image of the regions of ordered atoms on a surface marked with a square in Figure 1 is shown in Figure 2.

The EDS spectra obtained from dark nanostructures (not shown) contain the characteristic line AgLα = 2.98 keV, which allows attribute dark nanostructures to AgNPs. Most of AgNPs are in the form of chains and agglomerates, which is a characteristic feature of the systems prepared by MVS. The particle size distribution is narrow and monomodal. The average particle size is 1.75 ± 0.25 nm.

**Figure 3:**
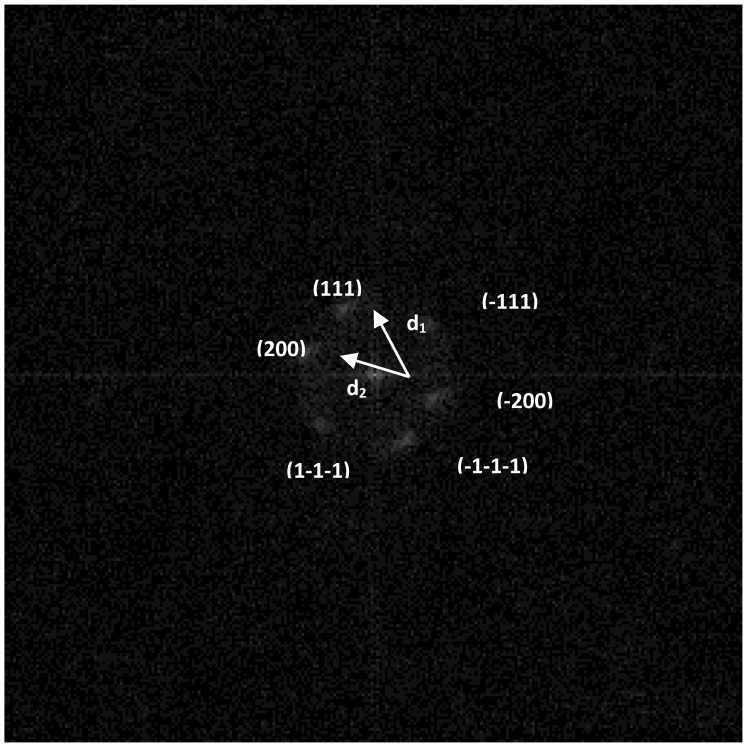
Simulation of the electron diffraction pattern for the group of atoms circled in Figure 2 and assignment for face-centered cubic (fcc) structure.

Simulation of the electron diffraction for the group of the atoms selected in Figure 2 is shown in Figure 3. It is seen from Figure 2 and Figure 3 that groups of atoms form faces with interplanar distances d1 and d2. Calculating the ratio d1/d2 gives a value of 1.09. The angle between d1 and d2 is 52°. The values 1.09 and 52° indicate the presence of faces (111) and (200) of a face-centered cubic (fcc) structure in silver particles (for an ideal fcc structure: d(111)/d (200) = 1.15, the angle is 55°).

Based on the synthesis method and sample storage conditions, three models of the chemical composition of the particle surface can be proposed: Ag^0^, Ag^+^, and Ag^0^ + Ag^+^. With a high degree of probability, the mixed composition in crystalline form can be excluded from the consideration. Otherwise, “double reflexes” should be observed in Figure 3. The fcc lattice constants (**a**) for Ag^0^ and Ag_2_O are 4.08 Å and 4.76 Å, respectively. Calculation of **a** by the formula **a** = d(hkl) × (h^2^ + k^2^ + l^2^)^1/2^, where h = 1, k = 1, l = 1; d (hkl) = 2.3 Å, gives a value of 3.98 Å. Consequently, there is reason to believe that the surface of the crystalline particles consists of metallic silver. However, presence of Ag^+^ state in amorphous form cannot be excluded. 

**Figure 4:**
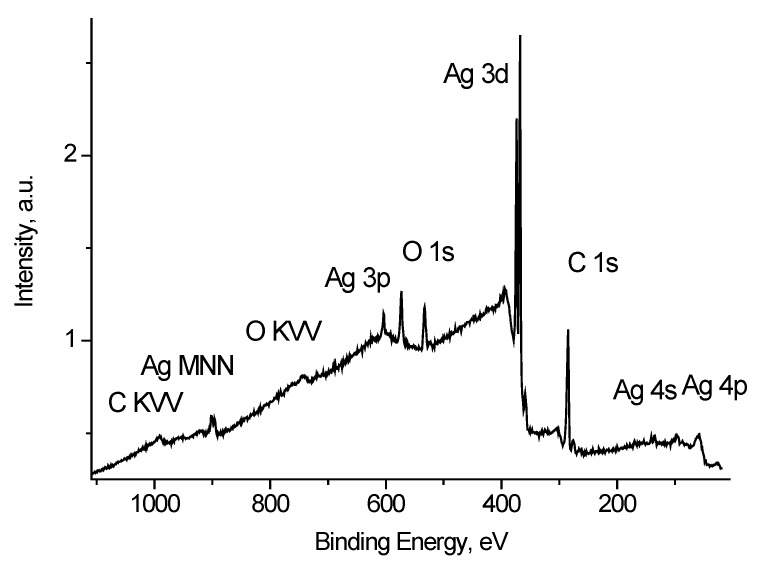
X-ray photoelectron spectroscopy (XPS) survey spectrum of Ag black.

Figure 4 shows the survey spectrum of Ag black. Along with the peaks characteristic of silver atoms there are peaks of impurity carbon and oxygen atoms.

The determination of the chemical state of silver atoms in nanoparticles by the XPS method is a complex task. This is due to the fact that the spectral characteristics of the metal particles and the oxide particles are fairly close. According to NIST XPS Database [14] the binding energies of the Ag 3d_5/2_ peak for Ag, Ag_2_O and AgO are in the ranges 367.9–368.4, 367.7–368.4, and 367.3–368.1 eV, respectively. One of the solutions to this problem is the use of the Auger parameter. However, as a rule, the concentration of silver nanoparticles in materials is low, whereas the registration of the Auger spectrum requires a significantly longer time than the recording of the photoelectron spectrum, which can lead to the reduction of silver from oxides under the action of X-ray irradiation [15].

Another approach may be based on controlled differential charging, in which separation of signals from regions with good and poor conductivity is possible. If the region has a metallic conductivity and is in good electrical contact with the sample holder, then it does not accumulate a charge due to the emission of secondary electrons. In contrast, in a region with poor conductivity, a positive charge is usually accumulated, the value of which depends on the secondary emission coefficient and conductivity, and it leads to the displacement of photoelectron peaks in the region of high binding energies. When a positive bias voltage U_b_ is applied, the stray electrons flow to the sample surface is increased and contributes to the compensation of the surface charge. In this case, narrowing of the photoelectron peaks and their shift toward higher binding energies are observed. Photoelectron peaks corresponding to regions with good conductivity should be shifted by U_b_ the amount of applied bias voltage, while from areas with a worse conductivity by a smaller amount. With a negative bias voltage, the flux stray electrons decreases, it leads to increase in charging on non-conducting regions and an increase in the interval between peaks corresponding to signals from the conducting and non-conducting regions. In this case, the signal from the conductive region must also be shifted by U_b_.

Taking into account the difference in the electrical conductivity of metallic silver and its oxides, we attempted to separate signals from regions containing silver atoms in a metal state and other chemical states by controlled differential charging, changing the potential on the sample holder. This approach is widely used to determine the presence of different phases in a sample [16,17,18,19,20,21].

**Figure 5:**
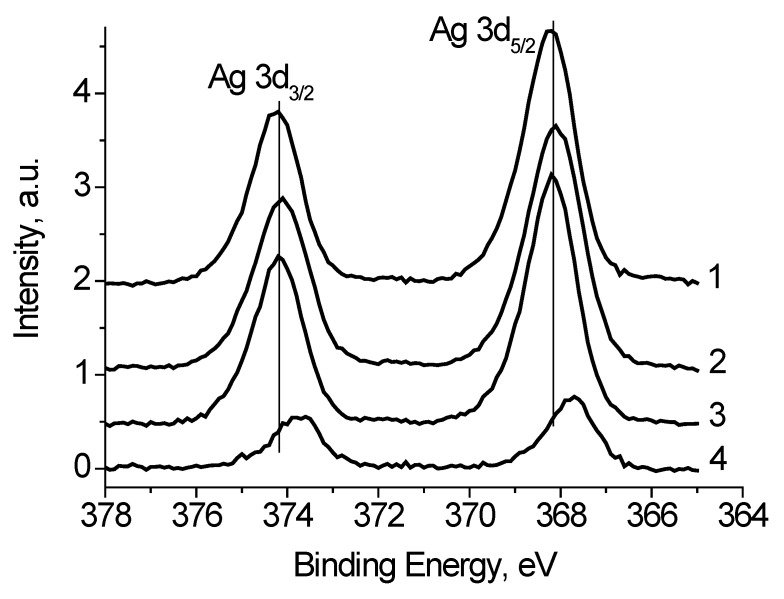
The Ag 3d photoelectron spectra of Ag black measured at different U_b_ = +7 (1), −7 (2), 0 V (3), ant difference spectrum (2)–(1). The spectra are corrected for U_b_.

To discriminate regions with different conductivities, or other words with different chemical states, a bias voltage U_b_ = ±7 V was supplied to the sample holder. Figure 5 shows the Ag 3d spectra of the Ag black measured at different bias voltage U_b_ applied to the sample holder. It is seen that binding energy of the Ag 3d_5/2_ and Ag 3d_3/2_ peaks is slightly depends on U_b_. Table 1 presents the characterization of the Ag 3d spectra. The full widths at high maximum (FWHM) are rather different as well. Both the binding energies of the Ag 3d peaks and their FWHM values indicate that the spectra contain some states with different conductivities. Considering that the recording the Ag_2_O and AgO spectra is accompanied by the surface charging [22,23], one can assume the presence of the Ag^+^ and/or Ag^2+^ state in the Ag black. To determine the characteristics of the Ag^δ+^ state a subtraction of the spectrum measured at U_b_ = 7 V from the spectrum measured at U_b_ = −7 V was performed under the condition of their best coincidence in the high-energy region. The difference spectrum is presented in Figure 5. The binding energies of the Ag 3d_5/2_ and Ag 3d_3/2_ peaks 367.73 and 373.71 eV correspond to Ag_2_O state [22]. The relative intensity of this state is no less than 0.27. This estimate is based on the fact that the spectrum measured at U_b_ = 7 V may contain Ag_2_O state.

**Figure 6:**
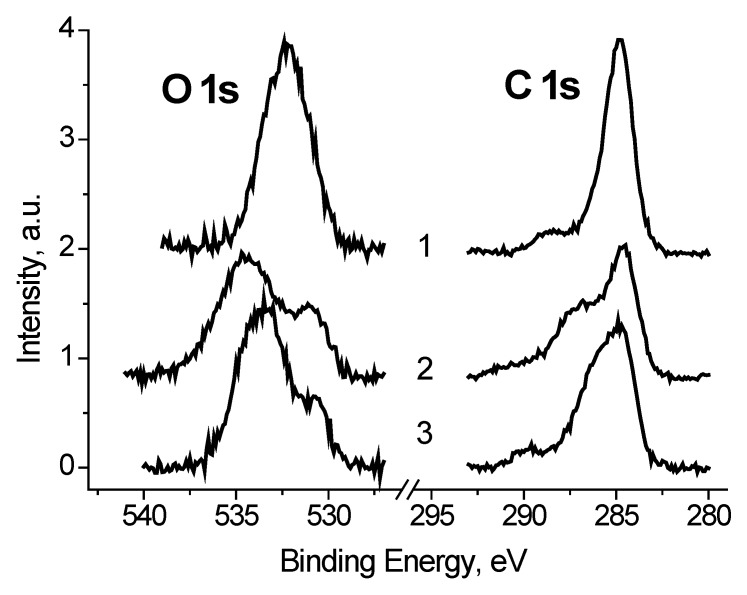
The C 1s and O 1s photoelectron spectra of Ag black measured at different U_b_ = +7 (1), −7 (2), 0 V (3).

The C 1s and O 1s spectra of Ag black show a strong dependence on U_b_ (Figure 6) which indicate that a large part of carbon and oxygen is has low conductivity. It should be noted that binding energy of the main C 1s peak measured at U_b_ = +7 V is 284.77 eV, and corresponds to that used for charge reference [14]. 

**Figure 7:**
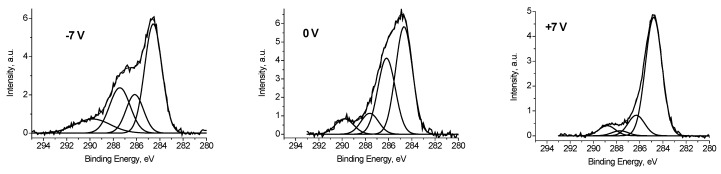
Fitting the C 1s photoelectron spectra of Ag black measured at U_b_ = −7, 0 and 7 V.

It follows that some of the carbon atoms have good conductivity, or in other words, is in close contact with silver atoms. To estimate this value, we use the fitting the C 1s spectrum measured at U_b_ = −7 V, when the best separation of the electron emission from regions with good and poor conductivity is realized. When the spectrum was fitted with some components, two restrictions were imposed: The width of the low-energy peak should describe the low-energy side by the best way, and the energy interval between the low-energy peak and the next should not be less than that at U_b_ = +7 V. The second restriction is imposed because of the possible manifestation of differential charging for regions containing C-O groups. The fraction of such carbon atoms is 0.47. A similar value of 0.48 was obtained for the spectrum measured at U_b_ = 0 V, whereas for the spectrum measured at U_b_ = +7 V it is 0.77. This is due to conductivity induced with the stray electrons, which make conducting regions that are not in direct contact with silver nanoparticles. Figure 7 shows fitting the corresponding C 1s spectra, and Table 2 contains corresponding data.

It should be noted that the fitting the C 1s spectra with some components measured at U_b_ = −7 and 0 V, presented in Figure 7 is largely conditional and do not reflect the real relative concentrations of CO_x_ groups. At the same time, applying a positive bias voltage U_b_ = +7 V practically compensates the surface charging, and Figure 7 (+7 V) reflects the real relative concentrations of CO_x_ groups.

**Figure 8:**
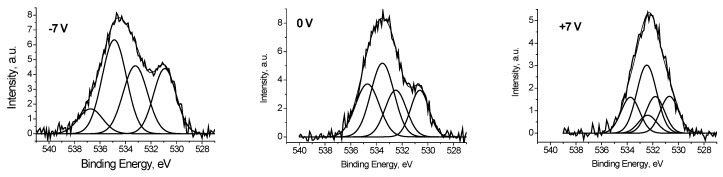
Fitting the O 1s photoelectron spectrum of Ag black measured at U_b_ = −7, 0 and 7 V.

It was found that the peaks in the low-energy region of the O 1s spectra, measured at U_b_ = −7 and 0 V (Figure 8), have close binding energies and intensities. Based on the reference data [21,22], the binding energy of these peaks of about 530.8 eV cannot be attributed to C-O bonds. Therefore, they should be attributed to the Ag-O bonds. And from the weak dependence of E_b_ and I_rel_ on U_b_, one can assign binding energies of 530.6 and 530.9 eV to Ag-Ag-O state. The I_rel_ of this state, determined from the fitting the O 1s spectra measured at U_b_ = −7, 0 and 7 V, are 0.24, 0.18 and 0.18, respectively.

It should be stressed that relative intensities of CO groups in the O 1s spectrum measured at U_b_ = 7 V correspond those obtained from the relative C 1s spectrum. It means that the bias voltage of U_b_ = 7 V neutralize the surface charging.

**Figure 9:**
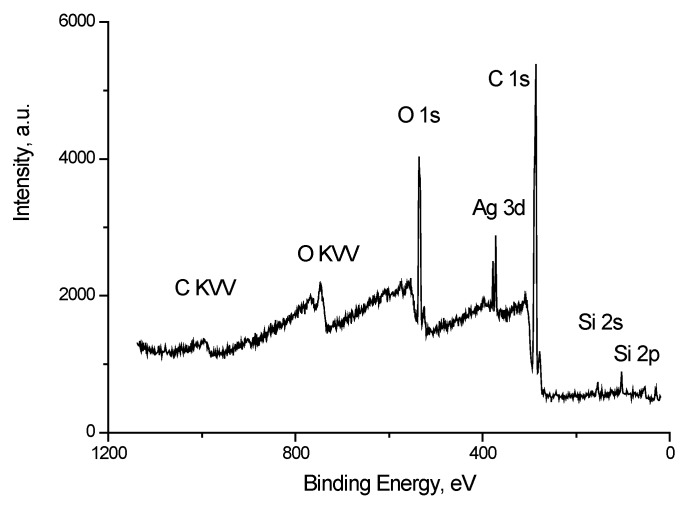
Survey spectrum of the Ag/bandage system.

Figure 9 shows the survey spectrum of the Ag/bandage system. Along with the peaks characteristic of silver, carbon and oxygen there are peaks of impurity silicon atoms.

**Figure 10:**
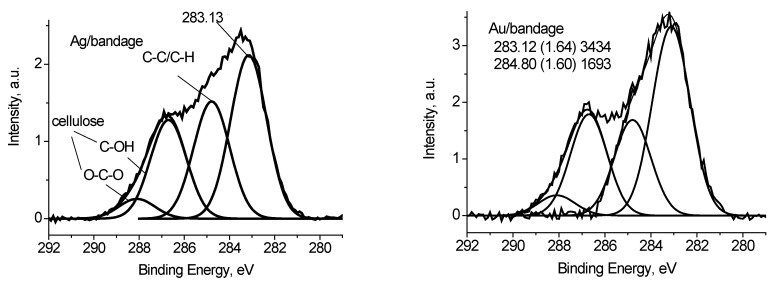
The C 1s photoelectron spectra of Ag/bandage and Au/bandage systems.

In case of the Ag/bandage system the charge referencing was done using the C 1s spectrum of cellulose. The latter was simulated using the reference data [24] by considering the difference in peak resolution. The binding energy of 286.7 eV was assigned to C-OH state of cellulose. Figure 10 shows the C 1s spectrum of the Ag/bandage sample fitted with four Gaussian peaks at 286.7, 288.1, 284.8 and 283.13 eV. The first and the second peaks are assigned to cellulose [24]. The third peak is assigned to adventitious carbon, while the origin of the peak at 283.1 eV is not clear because it does not correspond to reference data for polymers [24]. However, it may be resulted either of differential charging or low-molecular weight species. Similar С 1s spectrum was recorded for Au/bandage system. It slightly differs in relative concentration of C-C/C-H peak and peak at 283.1 eV. The O 1s spectra of Ag/bandage and Au/bandage systems are practically indistinguishable. It means that the peak at 283.1 eV may be assigned to C-C/C-H state as well.

**Figure 11:**
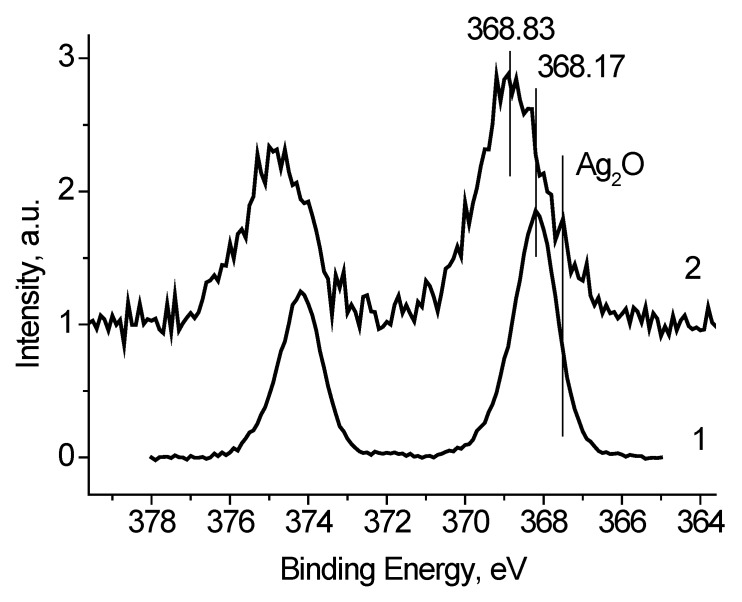
The C 1s photoelectron spectra of Ag black (1) and Ag/bandage system (2).

**Figure 12:**
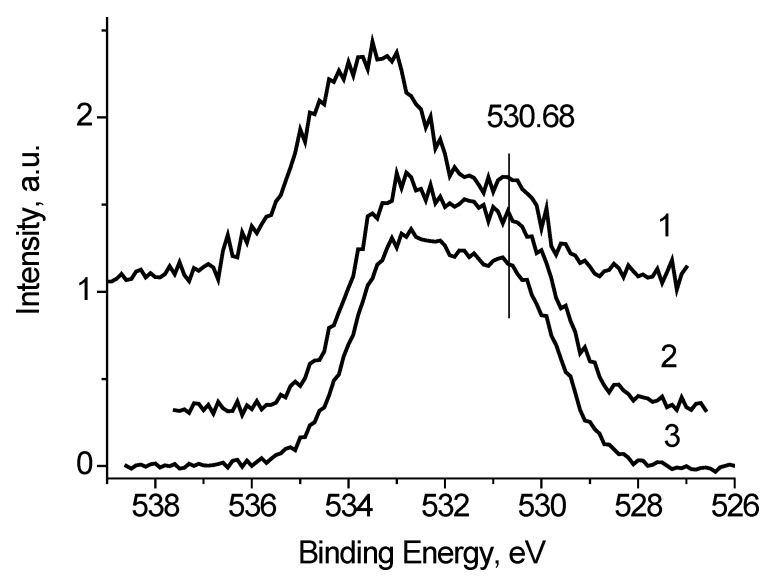
The O 1s photoelectron spectra of Ag black (1), Ag/bandage (2), and Au/bandage systems (3).

Figure 11 shows the C 1s spectra of Ag black and Ag/bandage system. It is clearly seen that the spectra are strongly different. The C 1s peak of the Ag/bandage system shifted to high binding energy region by 0.66 eV and its FWHM is 1 eV more than that of Ag black. These differences may be assigned to the size effect in photoelectron spectra which induces both energy shift to high binding energy and the peak broadening [25,26]. The transition from AgNPs in the Ag black, to their dispersion in the bandage followed with fairy large changes in size of AgNPs, and an increase of the proportion of the Ag^0^ state was observed. Apparently, there was a partial reduction of silver and its stabilization by a modified layer of cellulose. However, as follows from a comparison of the O 1s spectra of the Ag black and Ag/bandage system (Figure 12), the low-energy side of the latter might be assigned to the Ag-Ag-O group. 

But, given the almost complete coincidence of the O 1s spectra of Ag/bandage and Au/bandage systems, this conclusion should be rejected. Thus, one can conclude that the basic state of Ag atoms in Ag/bandage system is Ag^0^ state, whereas the oxidized silver is in the form of Ag-Ag-O groups, and, as follows from Figure 12 the proportion of oxidized state is small. This is in accordance with EXAFS and TEM data which indicate that silver atoms are mainly in Ag^0^ state. It should be noted that EXAFS is not a surface-sensitive method as XPS and electron diffraction may be recorded only from the ordered regions.

Table 3 shows the number of colony-forming units (CFU) of the studied microbes along the perimeter of the bandage at a distance equal to the diameter of one colony on both sides of the edge in the form Me (Q_1_; Q_3_), together with the level of statistical significance, where Me is the median, Q_1_—lower quartile, Q_3_—upper quartile.

Due to the fact that the data of the control groups of different strains differ, in order to compare the antibacterial effect of the AgNPs-containing bandage with respect to different microorganisms, we calculated the percentage reduction factor. Table 4 presents the results of the study of the percentage reduction of CFU.

The results of the change in the antibacterial properties of the ordinary medical gauze bandage under the influence of laser irradiation are presented in Table 5, in the form Me (Q_1_; Q_3_), where Me is the median, Q_1_ is the lower quartile, Q_3_ is the upper quartile.

Based on the data presented in Table 5, no statistically significant changes in the indices of the number of CFUs when using an ordinary medical gauze bandage without or in conjunction with laser irradiation have been identified. This indicates the absence of an antibacterial effect in laser radiation at a wavelength of 470 nm (blue region of the spectrum), a 5 mW power, and with exposure time of 5 min.

Table 6 shows the results of the change in the antimicrobial properties of the medical gauze bandage containing AgNPs, depending on the presence or absence of exposure to the laser and the time through which it was performed.

According to the data presented in Table 6, laser radiation in the blue region of the spectrum did not have a statistically significant increase in the antibacterial effect of AgNPs when applied two hours after inoculating the Petri dish and placing a bandage on it. However, when the laser treatment was used four hours after the seeding an increase in the antibacterial effect of this bandage was observed with respect to all studied microorganisms. The phenomenon is statistically significant in all groups of microorganisms. The difference can be observed in Table 7.

According to the data, presented in Table 7, there is no significant diversity between difference in percentage reduction of CFU of Gram-positive and Gram-negative microorganisms. Considering the fact that they differ in the structure of the cell wall, it can be concluded that the mechanism for increasing the antibacterial effect of the AgNPs-containing bandage cannot be explained solely by the effect on it. As follows from Table 7, laser radiation, when exposed four hours after inoculating the Petri dish and the application of a AgNPs-containing bandage on it allows to increase the antibacterial properties of the bandage by 15–24%, depending on the strain of the microorganism.

## 3. Discussion

It is believed that the antibacterial effect of silver ions is due primarily to the high affinity of the latter to sulfur or phosphorus [27]. Namely, it depends on the silver cations (Ag^+^), which bind firmly to groups of electron donors in biological molecules containing silver, oxygen or nitrogen. Due to the large number of sulfur-containing proteins on the surface of the bacterial cell, AgNPs can interact with sulfur-containing proteins inside or outside the cell membrane, which affect the viability of the bacterial cell [27]. Silver ions act by replacing other necessary metal ions, such as Ca^2+^ or Zn^+^ [28].

Simultaneously, it was suggested that silver ions (especially Ag^+^) liberated from AgNPs can interact with phosphorus moieties in DNA, leading to inactivation of DNA replication [27] or react with sulfhydryl groups of metabolic enzymes of the chain of bacterial transport of electrons, causing their inactivation [29].

The release of silver ions is only one of the mechanisms of AgNPs action. AgNPs themselves have different physical, chemical and other properties than the solid silver from which they were obtained.

A number of authors suggest that the antibacterial effect of silver nanoparticles is due to the electrostatic interaction between negatively charged bacterial cells and positively charged nanoparticles [30].

Analysis of the electronic state of AgNPs showed that the core of the particles is zero-valent, while the shell is oxidized by interaction with the functional groups of the bandage and the surrounding medium. Earlier, analysis of the O 1s and valence band spectra showed the existence of four states of oxygen atoms on the Ag surface, which reflect the different states of silver atoms as well [31]. The discrimination of these in the Ag 3d spectrum is practically impossible because of insufficient overall energy resolution of electron energy analyzers. The surface atoms of nanoparticles, which, as a rule, are positively charged, should play the main role in interaction with the bacterial membranes. The Ag 3d spectra shoe that a large fraction (0.27) of the Ag atoms is in the Ag^+^ state, which determines the antimicrobial effect. It is possible that laser radiation in the surface plasmon resonance mode changes the electronic state of AgNPs, which increases the antibacterial activity of the system as a whole.

The wavelength of laser radiation used in the study was chosen by considering the plasmon resonance effect. When the frequency of the external field coincides with the frequency of the localized surface plasmon, a resonance arises, leading to a sharp increase in the field on the surface of the particle and an increase in the absorption cross section [32].

Apparently, the statistically significant effect of enhancing the antibacterial properties of the medical gauze bandage containing AgNPs observed in the present study is caused by laser photothermolysis of bacteria when exposed to laser radiation.

Probably, AgNPs, when irradiated with a laser, absorb energy, which is transformed into heat. The resulting hyperthermia of AgNPs can lead to both local damage of the bacterial cell and intensification of metabolic processes around the heated AgNPs with the subsequent acceleration of silver ionization processes, which in turn leads to the death of the microbial cell.

## 4. Materials and Methods

The ordinary medical gauze bandage was used in the study, produced in the Republic of Belarus (State Standard 1172–93) both as a control and for the production of medical gauze bandages containing AgNPs.

**Scheme 1:**
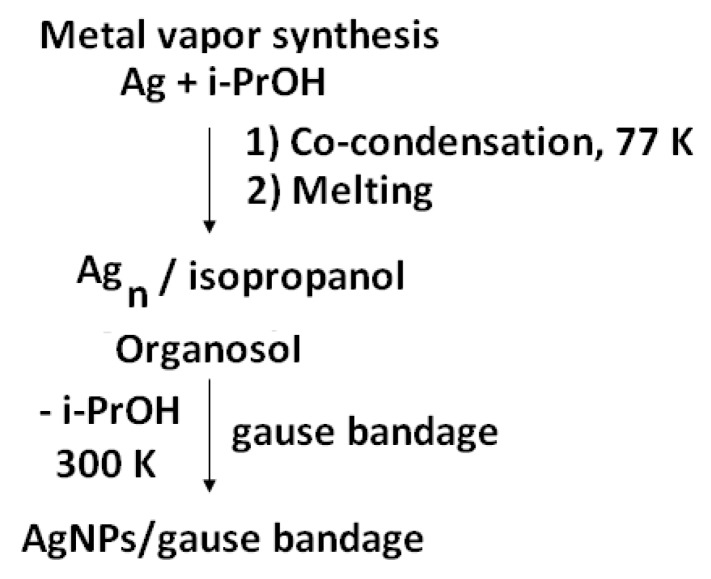
Synthesis of silver-containing nanocomposite.

The metal vapor technique was used for the preparation of Ag-bearing bandage (Scheme 1).

The silver nanoparticles were obtained by the MVS from pieces of silver (99.99%) using an apparatus described elsewhere [33,34,35,36]. In the preparation of silver organosol, isopropanol was used as the organic dispersion medium, which was degassed in the vacuum prior to synthesis by alternating freeze-thaw cycles. Isopropanol (Fluka, 99.8%) was dried over zeolites 4 Ǻ, and distilled under argon.

Silver was evaporated by resistive heating from a tantalum boat. During the synthesis, a vacuum of no more than 10^−2^ Pa was maintained in a 5-L quartz glass reactor with using a high-vacuum post. In a typical experiment, 120–150 mL of organic reagent was used in the synthesis and 0.1–0.12 grams of metal were evaporated. The supply of the organic reagent was adjusted with a fine adjustment valve. Before the synthesis, the glass reactor flask was evacuated, immersed in a vessel with liquid nitrogen, and then an organic reagent isopropanol was fed, which was condensed on the cooled walls of the reactor together with the metal vapors for about 1.5 h.

After the synthesis was completed, the cooling was stopped; the reactor was cut off from the vacuum post with a slide gate. Argon was fed to the reactor; the co-condensate of metal and organics was heated to the melting point. The obtained colloidal silver solution in isopropanol was impregnated with a bandage, which was before modification in a vacuum flask. The excess organosol was removed by drying in a vacuum of 1 Pa at a temperature of 80 °C.

Four strains of Gram-negative microbes (*Pseudomonas aeruginosa*, *Klebsiella pneumoniae*, *Escherichia coli*, *Moraxella* spp.) and two strains of Gram-positive bacteria (*Staphylococcus aureus*, *Staphylococcus haemolyticus*) were used. The strains were sowed from purulent wounds of patients of surgical departments in Grodno (Belarus).

The sampling for microbiological testing was performed in patients with purulent wounds using standard disposable sterile tampons by Heinz Herenz company, within an hour the material was delivered to a microbiological laboratory where a pure microbial culture was isolated and identified with a BioMerieux Vitek apparatus, antibiotic susceptibility of each microorganism was defined.

The bacteria sensitivity to the six most commonly used antibiotics in surgical hospitals in Belarus (amoxicillin (ACC), cephalexin (CFL), gentamicin (GEN), ciprofloxacin (CIP), cefazolin (CZ), erythromycin (ERI)) is presented in Table 8. Definition of antibacterial sensitivity was performed by diffusion into agar using discs.

Then, the isolated culture of the microbe was inoculated on sloping beef-extract agar, after 24 h cultivation, a sterile 0.85% solution of NaCl (5 mL) was flushed and diluted to the desired concentration with the same solution by successive inoculation into Petri dishes with agar of different concentrations of the microorganism. The desired concentration corresponded to the formation, after seeding, with a pipette, of 0.1 mL of the microbe suspension and placing the Petri dish in a 24 h thermostat for about 100 CFU. The following concentrations were used in the study: 0.5 × 10^−6^ for *Staphylococcus aureus*, 0.5 × 10^−5^ for *Staphylococcus haemolyticus*, *Klebsiella pneumoniae* and *Escherichia coli*, for *Pseudomonas aeruginosa* 1 × 10^−7^ and for *Moraxella* spp. 1 × 10^−6^.

The obtained suspension of microorganisms (0.1 mL) was seeded on a Petri dish with beef-extract agar. Then, two bands of medical gauze bandage were placed on each cup, measuring 1.5 × 4 cm. A standard medical gauze bandage was used as a control, medical gauze bandage containing AgNPs was used in the experimental groups. After that, all Petri dishes were placed in a thermostat at a temperature of 37.0 °C on 1 day for cultivation. After 24 h, CFU were counted on both sides of the edge of the bandage at a distance from the edge equal to the diameter of one colony ad oculus and using a binocular magnifier glass.

In addition, the percentage decrease in the number of CFU was calculated by the Formula (1):

Percentage reduction of CFU (%) = 100 × (A − B)/A(1)

where A is the average value of the number of CFU along the edge of the bandage in the control groups; and B is the average value of the number of CFU in the groups with medical gauze bandage containing AgNPs.

Microbial strains were cultivated on the Pronadisa beef-extract agar manufactured by Laboratorios Conda, S.A., which was prepared and sterilized according to the instructions of the manufacturer.

Sterilization of the experimental and control samples of the bandage was carried out by autoclaving at 121 °C during 16 min with a Cliniklav-25 vacuum autoclave.

Laser irradiation was performed by a Rodnik-1 therapeutic laser apparatus, a wavelength of 470 ± 30 nm (blue region of the spectrum), with a power of 5 mW, for 5 min. Laser irradiation was performed 2 and 4 h after inoculating the Petri dish and placing the dressing on it.

The schematic view of the irradiation experiment is shown on the Figure 12.

**Figure 13:**
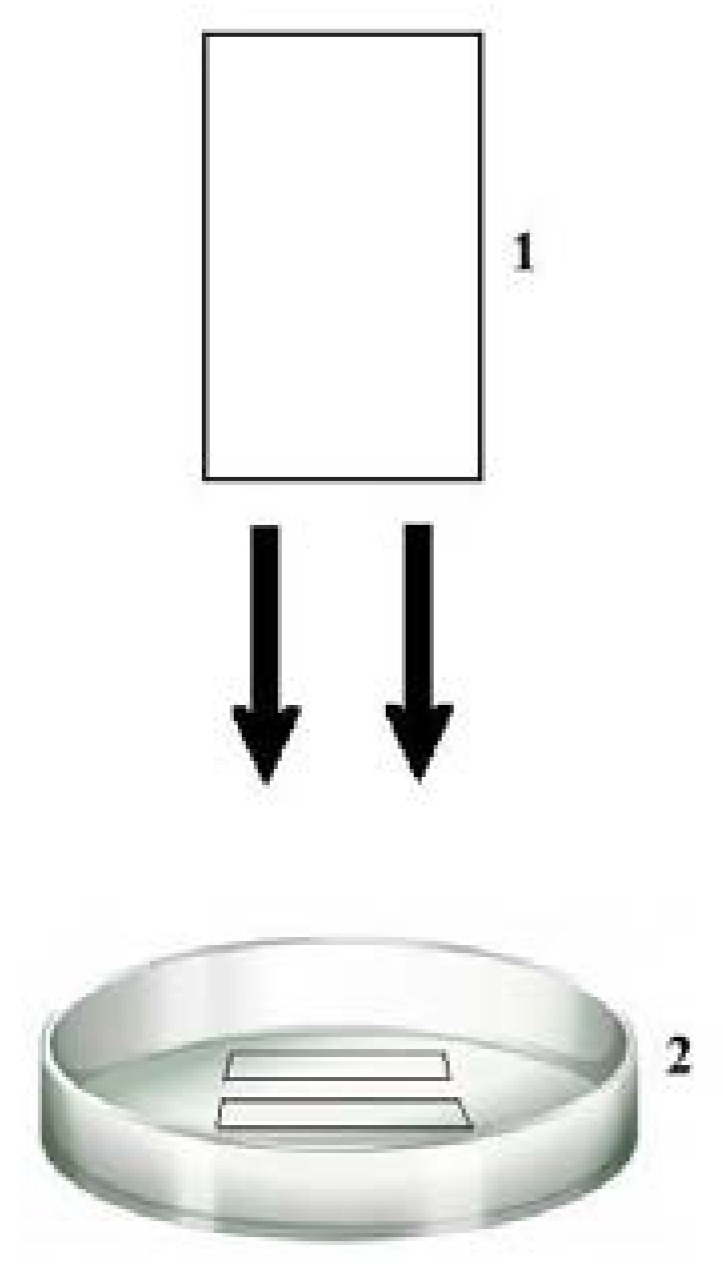
The scheme of the laser experiment; 1—laser and 2—two pieces of the bandage.

Figure 13 shows a Petri dish with agar, and two pieces of bandage placed on it irradiated with a laser.

The frequency selected in the study is determined by the frequency of plasmon resonance of AgNPs, the power and time of the exposure are determined experimentally.

Statistical processing of the results was carried out using the program Statistica 10.0. The difference between the groups was estimated using the nonparametric Mann-Whitney U-test at a given 5% significance level.

To assess the degree of increase in the antibacterial effect, the difference between the percentage decrease in the number of CFUs in the group where a AgNPs-containing bandage without laser irradiation was used and the percentage reduction in the group with laser irradiation was calculated 4 h after seeding the Petri dish and placing the bandage on it using the Formula (2):

Difference in the percentage reduction of CFU (%) = 100 × (A − B)/A = 100 × (A − C)/A(2)

where A is the average value of the number of CFU along the edge of the bandage in the control group (ordinary medical gauze bandage without laser irradiation); B is the average value of the CFU in the band with medical gauze bandage containing AgNPs with laser irradiation four hours after seeding the Petri dish and placing the bandage on it; and C is the average value of the number of CFUs in the group with medical gauze bandage containing AgNPs without laser irradiation.

Micrographs of the Ag-cotton sample were obtained by transmission electron microscopy (TEM) using a JEOL JEM 2100F/UHR instrument with a resolution of 0.1 nm. Prior to the test, 0.1 g of the sample was placed in 30 mL of isopropanol and sonicated for 300 s. A drop of the resulting mixture was placed on a carbon-coated copper TEM grid and dried for 1 h. The size of the Ag-containing particles was determined as the maximum linear dimension. To construct a histogram of the particle size distribution, the TEM data on 192 particles were processed. Identification of the chemical composition of the particles and the surface of the samples was carried out using an energy dispersive analysis (EDA) on a JED-2300 instrument. The interplanar distances in structures visible in high resolution TEM photographs were calculated using the electron diffraction patterns obtained with the fast Fourier transform in the ImajeJ 1.4 code. To assign the electron diffraction patterns to the crystallographic faces of the silver compounds, a crystallographic JCPDS database was used.

The X-ray photoelectron spectra were recorded using a XSAM-800 spectrometer (Kratos, Manchester, UK) with Mg Kα radiation at an operating power of 90 W of the X-ray tube in the fixed analyzer transmission mode at ~20 °C and a pressure in the analytical chamber of ~10^−8^ Pa. Survey and high-resolution spectra of appropriate core levels were recorded with step sizes of 1 and 0.1 eV, respectively. The photoelectron spectra were approximated by Gauss function or the sum of Gauss functions, and the background caused by secondary electrons and photoelectrons that lost energy, was approximated by the straight line. The energy scale of spectrometer was calibrated according to the standard procedure, while considering the following binding energies: 932.7, 368.3, and 84.0 eV for Cu2p_3/2_, Ag3d_5/2_, and Au4f_7/2_, respectively. Quantification was performed using atomic sensitivity factors included in the software of the spectrometer. The samples were fixed to the sample holder by double-sided conductive adhesive tape. Sample charging was corrected by referencing to the C-C/C-H state deconvoluted in the C ls spectrum (284.8 eV).

## 5. Conclusions

1. The Ag-cotton nanocomposites synthesized via MVS has a pronounced antimicrobial activity against some Gram-positive and Gram-negative bacteria.

2. Antibacterial properties of medical gauze bandage containing AgNPs significantly increase when exposed to laser radiation. The effect of enhancing the antibacterial properties of the nanocomposite apparently associated with the effect of localized plasmon resonance of AgNPs.

3. TEM data indicate that the particle size distribution is narrow and monomodal with average particle size of 1.75 nm. EDA shows that interplanar distances correspond to metallic nanoparticles. XPS and EXAFS demonstrate that Ag atoms in Ag/bandage system is Ag^0^, the oxidized silver is in the form of Ag-Ag-O groups. 

## Figures and Tables

**Table 1:** Binding energies and full widths at high maximum (FWHMs) measured by XPS.

U_b_	Ag 3d_5/2_, eV	Ag 3d_3/2_, eV	Ag 3d_5/2_ FWHM, eV
0 V	368.17	374.17	1.3
−7 V	368.09	374.11	1.4
+7 V	368.21	374.21	1.5
(−7 V)–(+7 V)	367.73	373.71	1.3

**Table 2:** Binding energies (E_b_), peak widths (W) and relative intensities of the peaks deconvoluted in the C 1s and O 1s spectra of Ag black.

Ub		C 1s				O 1s				
		C-C/C-H	C-OH/C-O-C	C=O	C(O)O	Ag-O	C=O/C(O*)O	C-OH/C-O-C	C(O*)O	C(O)O*
0 V	E_b_	284.7	286.2	287.6	289.8	530.6	532.5	533.5		534.7
	W	1.45	1.50	1.51	1.58	1.42	1.65	1.80		1.85
	I_rel_	0.48	0.35	0.10	0.07	0.18	0.21	0.35		0.26
−7 V	E_b_	284.6	286.2	287.4	289.8	530.9	533.2	534.9		536.7
	W	1.48	1.50	1.76	3.05	1.76	1.90	1.90		1.90
	I_rel_	0.47	0.17	0.23	0.13	0.24	0.27	0.38	0.00	0.10
+7 V	E_b_	284.8	286.3	287.7	288.8	530.7	532.4	532.5	531.8	533.8
	W	1.43	1.43	1.43	1.43	1.42	1.42	1.55	1.42	1.55
	I_rel_	0.77	0.13	0.03	0.07	0.18	0.09	0.37	0.18	0.18

**Table 3:** The number of colony-forming units (CFU) of the studied microorganisms along the edge of the bandage at a distance to both sides of the edge equal to the diameter of one colony (Me (Q_1_; Q_3_)) and the level of statistical significance (*p*) between control groups and gauze with AgNPs.

Strain of Microorganism	Control (Normal Bandage)	AgNPs-Containing Bandage	*p*
*Staphylococcus aureus*	7.0 (6.0; 8.0)	0.0 (0.0; 1.0)	<0.001
*Staphylococcus haemolyticus*	11.0 (7.5; 14.5)	7.5 (6.0; 8.0)	0.049
*Pseudomonas aeruginosa*	5.0 (5.0; 6.0)	2.0 (1.0; 3.0)	<0.001
*Klebsiella pneumoniae*	6.5 (6.0; 7.0)	2.0 (1.0; 3.0)	<0.001
*Escherichia coli*	16.0 (13.5; 17.5)	6.0 (4.0; 7.0)	<0.001
*Moraxella* spp.	8.0 (5.0; 8.5)	3.0 (2.5; 4.0)	0.002

**Table 4:** Percentage reduction in the number of CFU towards control.

Microbial Strain	Percentage Reduction of CFU of the AgNPs-Containing Bandage
*Staphylococcus aureus*	95
*Staphylococcus haemolyticus*	34
*Pseudomonas aeruginosa*	58
*Klebsiella pneumoniae*	68
*Escherichia coli*	64
*Moraxella* spp.	55

**Table 5:** The number of CFU of the investigated microorganisms along the edge of the ordinary medical gauze bandage (Me (Q_1_; Q_3_)) and *p*—the level of statistical significance between groups.

Strain of Microorganism	Control (Normal Bandage)				
	Group 1	Group 2	*p*1	Group 3	*p*2
	Without Laser Irradiation	Laser Irradiation after 2 h		Laser Irradiation after 4 h	
*Staphylococcus aureus*	6.0 (5.5; 7.0)	6.5 (5.0; 9.0)	0.682	6.5 (6.0; 8.0)	0.325
*Staphylococcus haemolyticus*	6.5 (5.5; 8.0)	7.0 (6.0; 8.0)	0.681	6.0 (4.0; 9.5)	0.620
*Pseudomonas aeruginosa*	14.0 (11.5; 15.5)	14.0 (13.0; 15.5)	0.861	14.5 (13.0; 16.5)	0.620
*Klebsiella pneumoniae*	5.0 (3.0; 5.0)	5.0 (4.0; 7.0)	0.223	4.0 (4.0; 5.0)	0.862
*Escherichia coli*	10.0 (9.0; 10.5)	10.0 (8.0; 12.0)	0.861	11.0 (8.5; 11.5)	0.380
*Moraxella* spp.	7.0 (4.0; 8.0)	6.5 (4.5; 8.0)	0.930	6.0 (5.0; 8.5)	0.838

*p*1 = the level of statistical significance between groups 1 and 2, and *p*2 = the level of statistical significance between groups 1 and 3.

**Table 6:** The number of CFU of the investigated microorganisms along the edge of the medical gauze bandage containing AgNPs, (Me (Q_1_; Q_3_)) and *p*—the level of statistical significance between the groups.

Strain of Microorganism	AgNPs-Containing Bandage				
	Group 4	Group 5	*p*1	Group 6	*p*2
	Without Laser Irradiation	Laser Irradiation after 2 h		Laser Irradiation after 4 h	
*Staphylococcus aureus*	5.0 (3.5; 5.5)	5.0 (4.0; 5.0)	0.904	4.0 (2.0; 5.0)	0.043
*Staphylococcus haemolyticus*	5.0 (4.0; 6.0)	5.5 (2.5; 6.5)	0.861	4.0 (2.5; 4.5)	0.011
*Pseudomonas aeruginosa*	7.0 (5.5; 8.0)	6.0 (5.0; 7.5)	0.250	4.0 (2.5; 5.0)	<0.001
*Klebsiella pneumoniae*	2.0 (1.5; 3.0)	2.0 (1.0; 3.0)	0.903	1.0 (1.0; 1.5)	0.016
*Escherichia coli*	6.5 (6.0; 7.0)	7.0 (5.5; 8.0)	0.727	5.0 (4.5; 6.0)	0.024
*Moraxella* spp.	3.5 (2.0; 4.0)	3.0 (2.0; 4.0)	0.723	2.0 (1.0; 3.0)	0.039

*p*1 = the level of statistical significance between groups 4 and 5, and *p*2 = the level of statistical significance between groups 4 and 6.

**Table 7:** Difference in percentage reduction of CFU between the groups of the AgNPs-containing bandage without laser irradiation and with laser irradiation four hours after inoculating the Petri dish and placing the bandage.

Strain of Microorganism	Difference in Percentage Reduction of CFU, %
*Staphylococcus aureus*	20
*Staphylococcus haemolyticus*	24
*Pseudomonas aeruginosa*	22
*Klebsiella pneumoniae*	22
*Escherichia coli*	15
*Moraxella* spp.	19

**Table 8:** Antibacterial sensitivity of the strains of microbes used in the study.

Microbial Strain	Antibiotics					
	ACC	CZ	CFL	CIP	GEN	ERI
*Staphylococcus aureus*	S	S	S	S	S	R
*Staphylococcus haemolyticus*	R	S	R	S	S	S
*Pseudomonas aeruginosa*	R	R	R	R	R	R
*Klebsiella pneumoniae*	R	R	R	R	R	R
*Escherichia coli*	R	S	S	S	S	R
*Moraxella* spp.	R	R	R	R	R	I

S = the microorganism is sensitive to this antibiotic, I = moderate resistance of the microorganism to the antibiotic presented, R = the microorganism is resistant to this antibiotic. ACC = amoxicillin, CFL = cephalexin, GEN = gentamicin, CIP = ciprofloxacin, CZ = cefazolin, ERI = erythromycin.
